# Eye-specific detection and a multi-eye integration model of biological motion perception

**DOI:** 10.1242/jeb.247061

**Published:** 2024-06-26

**Authors:** Massimo De Agrò, Daniela C. Rößler, Paul S. Shamble

**Affiliations:** ^1^ Faculty of Biology, University of Regensburg, 93053 Regensburg, Germany; ^2^ Department of Biology, University of Florence, 50121 Firenze, Italy; ^3^ The BioRobotics Institute, Sant'Anna School of Advanced Studies, 56127 Pisa, Italy; ^4^ Zukunftskolleg, Konstanz University, 78464 Konstanz, Germany; ^5^ Department of Biology, Konstanz University, 78464 Konstanz, Germany; ^6^ Department of Ecology of Animal Societies, Max Planck Institute of Animal Behavior, 78464 Konstanz, Germany; ^7^ Kavli Institute for Neuroscience, Department of Neuroscience, Yale University School of Medicine, New Haven, CT 06510, USA

**Keywords:** Jumping spider, Psychophysics, Life detector, Invertebrate, Vision

## Abstract

‘Biological motion’ refers to the distinctive kinematics observed in many living organisms, where visually perceivable points on the animal move at fixed distances from each other. Across the animal kingdom, many species have developed specialized visual circuitry to recognize such biological motion and to discriminate it from other patterns. Recently, this ability has been observed in the distributed visual system of jumping spiders. These eight-eyed animals use six eyes to perceive motion, while the remaining two (the principal anterior medial eyes) are shifted across the visual scene to further inspect detected objects. When presented with a biologically moving stimulus and a random one, jumping spiders turn to face the latter, clearly demonstrating the ability to discriminate between them. However, it remains unclear whether the principal eyes are necessary for this behavior, whether all secondary eyes can perform this discrimination, or whether a single eye-pair is specialized for this task. Here, we systematically tested the ability of jumping spiders to discriminate between biological and random visual stimuli by testing each eye-pair alone. Spiders were able to discriminate stimuli only when the anterior lateral eyes were unblocked, and performed at chance levels in other configurations. Interestingly, spiders showed a preference for biological motion over random stimuli – unlike in past work. We therefore propose a new model describing how specialization of the anterior lateral eyes for detecting biological motion contributes to multi-eye integration in this system. This integration generates more complex behavior through the combination of simple, single-eye responses. We posit that this in-built modularity may be a solution to the limited resources of these invertebrates' brains, constituting a novel approach to visual processing.

## Teaser

**Highlighted Article:** Jumping spiders discriminate biological from random motion using only one eye pair: a model of how the contribution of different eye specializations may produce emergent and more complex visual abilities is proposed.

## INTRODUCTION

Many animals have photosensitive cells that allow them to capture visual information from their environment (Lazareva et al., 2012). However, light collection is not always enough for more sophisticated visual tasks. Instead, patterns of activation need to be organized and interpreted, correctly assessing the current situation to inform subsequent decision making (DiCarlo et al., 2012). Because of the wide range of visual information types, this is a complex task. In humans and other vertebrates, this has driven the evolution of massive neural networks that use hierarchical processes to interpret the visual scene (Felleman and Van Essen, 1991; Hubel and Wiesel, 1962; Serre, 2014; Van Essen et al., 1992). Indeed, enlarging the brain through increased neuronal investment, as in humans, seems to be an effective response to the challenges presented by the complexity of the visual scene (Hofman, 2014). However, this strategy is not viable for smaller animals, which face similar visual tasks but lack the spatial capacity for brain growth. Arthropods seem to have found a solution. Behavioral evidence suggests that they, too, are capable of complex behaviors, despite their comparatively small nervous systems (Chittka and Niven, 2009; Eberhard, 2007, 2011; Eberhard and Wcislo, 2011; Goté et al., 2019) – including behaviors comparable to those of vertebrates, such as conceptual and multi-modal learning (Avarguès-Weber and Giurfa, 2013; Avarguès-Weber et al., 2012; De Agrò et al., 2020), or complex navigation (Wehner, 2003).

**Fig. 1.**
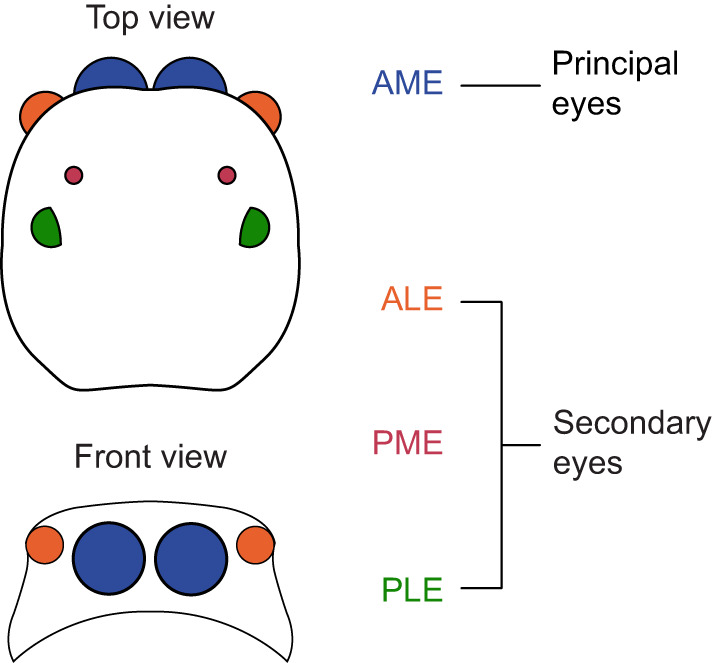
**Schematic representation of the eight eye pairs of the jumping spider.** Top and front views are shown. The anterior medial eyes (AMEs) are referred to as principal eyes and are movable, with a small visual field of around 5 deg each, but with high spatial resolution and color vision. These eyes are likely specialized for static figure discrimination. The other three pairs of eyes, anterior lateral eyes (ALEs), posterior medial eyes (PMEs) and posterior lateral eyes (PLEs) are referred to as secondary eyes. With a wider visual field but lower acuity, they are considered to be specialized for motion perception and discrimination. PMEs are considered to be vestigial in most species.

Jumping spiders show an impressive level of cognitive and behavioral complexity (Nelson, 2023). These include learning (De Agrò, 2020; De Agrò et al., 2017; Liedtke and Schneider, 2014; Mannino et al., 2023), numerical abilities (Cross and Jackson, 2017), spatial and action planning (Cross and Jackson, 2015, 2016, 2019; Tarsitano and Jackson, 1994), object recognition (Dolev and Nelson, 2014, 2016; Rößler et al., 2022a), and even engaging in REM sleep-like behaviors (Rößler et al., 2022b). Remarkably, the most distinctive feature of these animals is their vision (Winsor et al., 2023, 2024): a modular, specialized system organized into four pairs of eyes (Fig. 1), with each pair projecting into anatomically separate brain areas (Harland et al., 2012; Morehouse, 2020; Morehouse et al., 2017; Steinhoff et al., 2017, 2020). The two largest, forward-facing, anterior medial eyes (AMEs, principal eyes; Fig. 1) have the highest visual acuity and a layered retina that allows for single-eye depth perception (Nagata et al., 2012) and color vision (Land, 1969b; Zurek et al., 2015). These eyes have a narrow visual field (∼5 deg) and are moved by sets of muscles (Land, 1969a), achieving a function similar to the fovea in human eyes. The remaining three pairs of eyes – the anterior lateral eyes (ALEs), posterior median eyes (PMEs) and posterior lateral eyes (PLEs) – collectively referred to as secondary eyes (see Fig. 1), are monochrome and smaller. However, they boast a significantly wider visual field, covering a combined range of ∼350 deg. The principal and secondary eyes are thought to divide the labor of visual computation (Strausfeld and Barth, 1993; Strausfeld et al., 1993), with the primary eyes handling static detail while the secondary eyes specialize in perceiving movement. As soon as a stimulus is detected, the spider rapidly pivots to face the object with the principal eyes (Beydizada et al., 2024; Ferrante et al., 2023 preprint; Zurek and Nelson, 2012b; Zurek et al., 2010). The AMEs can then start scanning the target to inform object classification and recognition (Dolev and Nelson, 2014, 2016; Land, 1969a; Menda et al., 2014; Rößler et al., 2022a; Zurek and Nelson, 2012a).

The jumping spiders' secondary eyes are not limited to detecting moving objects but can also recognize different types of motion and can inform subsequent behaviors accordingly (Bruce et al., 2021; De Agrò et al., 2021; Spano et al., 2012). This ability extends to complex dynamic visual stimuli. In a previous experiment (De Agrò et al., 2021), we demonstrated that jumping spiders can recognize biological motion – a term that refers to stimuli that move according to a pattern common across virtually all living organisms (Johnson, 2006). During experiments, these stimuli are typically presented as clouds of dots, mapping the major joints of an animal during locomotion. However, these patterns lack any geometrical structure (Johansson, 1973, 1976; Lemaire and Vallortigara, 2022; Neri et al., 1998; Troje, 2013; Troje and Westhoff, 2006).

To date, it remains unclear whether the AMEs are necessary for processing these biological motion cues. Moreover, it is still unknown whether all secondary eyes can perform this discrimination or whether it is specialized to a single eye-pair. We hypothesize that as a result of the selection for functional specialization in the visual systems of jumping spiders, the detection of biological motion occurs in a single eye-pair rather than being distributed across multiple eyes and brain areas. Most likely, the eye-pair specialized in the task is the ALEs, given their motion-sensitive and forward-facing nature, in contrast with AMEs (not specialized in motion perception) and PLEs (backward facing, less useful for specific target detection). It is important to note that to discriminate biologically moving stimuli from randomly moving ones, analyzing a single dot trajectory is insufficient; instead, one must integrate the relative motions of multiple entities. In humans and other vertebrates, this complex integration is carried out by late visual areas, such as the medial temporal area, MT (Grossman and Blake, 2002; Grossman et al., 2000), yet spiders lack any brain area homologous or otherwise comparable to these structures. If discrimination in jumping spiders can occur in a single eye-pair, this would suggest that the computation happens in a dedicated brain area, very early in the visual stream. This ‘early’ rather than ‘late’ differentiation of biological motion cues would suggest a fundamentally different neural strategy and the presence of a currently unknown process for detecting biological motion.

To test this hypothesis, we selectively covered jumping spiders' eyes to leave them with only ALEs, only PLEs, or both pairs of secondary eyes un-occluded. We then presented them simultaneously with biological and random motion stimuli and recorded which stimuli they turned towards.

## MATERIALS AND METHODS

### Subjects

For the experiments, we collected jumping spiders of the species *Menemerus semilimbatus* (Hahn 1829) from the field. These spiders can be found in parks and on buildings, and are abundant across southern Europe. For the first experiment, 31 individuals were used (9 males, 14 females, 8 juveniles) while the second, main experiment involved 179 spiders (15 males, 89 females, 75 juveniles). Only animals with a body length exceeding 5 mm were collected to ensure the efficacy of the methodology. Once caught, the animals were housed in transparent plastic boxes (dimensions 80×65×155 mm). They were fed *Drosophila* fruit flies *ad libitum*, replenished once a week, until the time of the experiment.

### Eye treatment

The day before testing, a magnet was fixed to the prosoma (head) of each subject using UV glue (sculokic) in order to attach the animal to the treadmill apparatus (see De Agrò et al., 2021). During this procedure, we also covered the spider's eyes according to their assigned experimental treatment. Each spider was assigned to one of following three treatments: (i) ALE treatment – the animal had only their ALEs uncovered, as paint was applied over the AMEs, PMEs and PLEs; (ii) PLE treatment – the animal had only their PLEs uncovered, with paint applied over the AMEs, ALEs and PMEs; (iii) ALE+PLE treatment – the animal had the ALEs, PMEs and PLEs uncovered, with paint applied only over AMEs. We did not include a PME condition as PMEs are considered vestigial and with a limited field of view (Land, 1985; see Fig. 1 for eye organization).

Spider eyes were painted under the microscope using a toothpick with a small dab of water-based white paint. White paint was chosen over other colors for its visibility on the dark-colored spider eyes. Following the completion of all assigned trials for each animal, the magnet was removed, and the paint was washed off. The spider was then released in the same spot where it was captured. Magnets did not appear to negatively affect the animals during the short period in which they were housed in the lab, and spiders freed from the magnet appeared to move and behave normally.

### Experimental apparatus

**Fig. 2.**
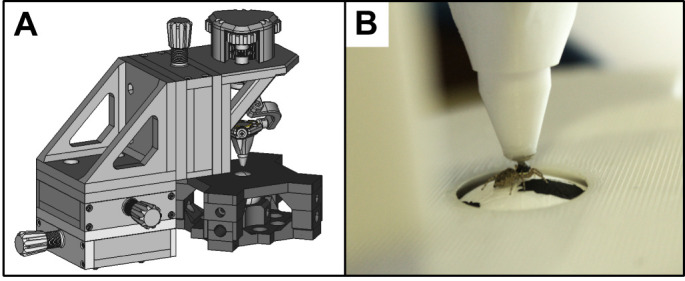
**Experimental apparatus.** (A) A 3D model of the 6-axis manipulator (light gray), and the sphere holder (dark gray). (B) Magnified image of a spider on the apparatus. The animal is connected to the end effector of the manipulator by a magnet. The legs are in contact with the visible section of the polystyrene sphere.

The experimental apparatus, stimuli and scoring used in these experiments were as described in De Agrò et al. (2021), except that the computer monitors were arranged differently (see below). In brief, a polystyrene sphere (38 mm diameter) was contained in a plastic holder, suspended by a constant stream of compressed air, and positioned in the center of the apparatus. The top of the plastic holder was open, revealing a 20 mm wide cap of the sphere below. At the start of each experiment, a spider was attached to the end effector of a 6-axis micro-manipulator using the magnet glued on top of the prosoma (Fig. 2A). Then, the animal was lowered and positioned with its legs in contact with the polystyrene sphere and oriented relative to the monitors. This way, despite being tethered, the spider was able to affect motion of the sphere below (Fig. 2B). By recording the sphere using a high-speed camera (120 frames s^−1^), we extracted the frame-by-frame rotational matrices (Moore et al., 2014) and thus the intended motion of the spider.

Two computer monitors (BenqGW2270, 1920×1080 pixels, 537 mm wide) were placed in front of the animal, angled towards each other at 120 or 65 deg, depending on the experimental condition (see below). The contact point between the two monitors was situated directly in front of the animal, at the center of their visual field, from here on defined as 0 deg. In both experiments, stimuli appeared on one or both of the monitors, moving from the outermost section towards the center, or vice versa. When detecting a stimulus with the secondary eyes, jumping spiders performed full-body pivots (Land, 1972), to focus the visual fields of the AMEs on the target. When presented with opposing information – for example, stimuli on two different sides – the spider preferentially turned towards one depending on valence, preference or possibly other factors (De Agrò et al., 2021).

To infer the spider's rotation, the frame-by-frame rotation of the sphere was extracted using the software FicTrac (Moore et al., 2014). We then focused on the sphere rotations around its *z*-axis, as they correspond to the spider's pivots. Using a custom script written in Python 3 (Van Rossum and Drake, 2009), and the packages *pandas* (doi:10.5281/zenodo.3509134, doi:10.25080/majora-92bf1922-00a), *numpy* (Harris et al., 2020) and *scipy* (Virtanen et al., 2020), we detected peaks in the signal, corresponding to probable rotation events. Positive and negative peaks were recorded, as they correspond to clockwise and counterclockwise rotations, respectively. Each peak could then be associated with the position of the stimulus based on the time of stimulus appearance and the recorded time of the peak. The full script is available in Supplementary Materials and Methods.

### Experiment 1 – identification of eye-specific visual angles

In the first experiment, we used a behavioral procedure to identify the extent of the visual field of every eye-pair in *Menemerus semilimbatus.* While information regarding eye-specific visual field spans is available for certain jumping spider species in the literature (Land, 1985; Zurek and Nelson, 2012a), visual fields can vary considerably across different species of Salticidae (Land, 1985). To draw this visual field map, we exploited the spiders' typical secondary eye detection behavior described in the Introduction: as these animals tend to perform a pivot immediately when a moving stimulus is detected, an object moving horizontally will trigger a reaction from the spider as soon as it enters its visual field. By recording the angular position of the stimuli upon first detection, we can behaviorally draw the edges of every eye.

**Fig. 3.**
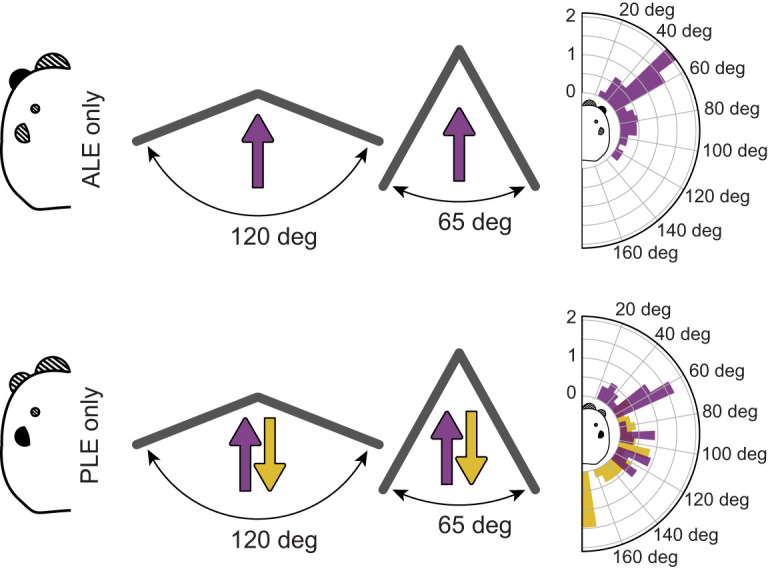
**Procedure and results for both conditions of experiment 1.** In the ALE-only condition, spiders were placed on two different setups: facing the meeting point of two monitors, angled 120 deg or 65 deg relative to each other either. Facing direction is indicated by the purple arrow. The plot on the right reports the relative frequency of saccades (*y*-axis) per position (angle) of the moving stimulus (*x*-axis). A clear peak in rotation frequency is apparent at 50 deg. In the PLE-only condition, the spiders were placed in the same two setups, but they could be oriented either towards the meeting point (purple) or away from it (gold). This was done so that stimuli could be presented all around the animal, rather than only in the front. In the resulting graph on the right, purple bars are for trials where the spiders were oriented towards the screens, gold bars are for when the spiders were oriented backwards (the graph is still represented from the spider's point of view, hence the bars are at the spider's back). When oriented forwards, a clear peak is visible at 60 deg. When oriented backwards, the peak is visible at 180 deg.

To present stimuli across the entire 360 deg around the animal, we varied the placement of the monitors and of the spiders throughout the experiment (Fig. 3). First, the two monitors were placed angled towards each other either at 120 or 65 deg. The spider was positioned at a distance of 200 mm from the contact point of the two monitors. The distinct positioning of the monitors naturally resulted in a varied coverage of the spider's visual field, with the first option spanning ∼200 deg and the second ∼265 deg. To account for the remaining ∼95 deg at the back of the spider, we reversed the spider's orientation, causing them to face away from the meeting point of the monitors (refer to Fig. 3).

Each spider in the PLE treatment (*n*=16) underwent four conditions across four randomly ordered trials, one for each setup (monitors at 120 deg, frontally facing; monitors at 65 deg, frontally facing; monitors at 120 deg, backward facing; monitors at 65 deg, backward facing; Fig. 3). Spiders in the ALE treatment (*n*=15) instead underwent two conditions, corresponding to the two frontally facing setups (Fig. 3), as the ALE visual field is generally identified as being ±50 deg (Zurek and Nelson, 2012a). No ALE+PLE spider underwent experiment 1, as the combined visual field would have been non-informative.

At the start of each trial, a spider was positioned on the polystyrene sphere, oriented according to the given trial and condition. After 210 s of habituation, a 4 deg wide circular stimulus appeared on either the left or the right monitor, vertically in line with the spider position (0 deg elevation), starting from either the contact point of the two monitors or the outer border. The stimulus then moved at 9 deg s^−1^ (the characteristics previously shown to trigger the highest saccadic probability; see Zurek et al., 2010), either towards the center of the visual field or away from it, and continuing until it reached the opposing edge of the monitor. A new stimulus appeared 15 s later, for a total of 30 presentations. For each presentation, we recorded the first pivot produced by the spider, and noted the angular position of the stimulus at pivot initiation.

For this experiment, we were interested in the first pivot performed by the spider, which most likely indicates when the stimuli first enter the animal's visual field. Therefore, we selected the first measured peak with a rotation minimum of 20 deg (determined by calculating the area under the curve for the selected signal peak) in the direction of the stimulus (clockwise for stimuli on the left, counterclockwise for stimuli on the right; note that the spider's intended rotation is opposite to the rotation of the sphere). We then recorded the position of the stimulus at the time of the first rotation and saved it as the point of detection.

### Experiment 2 – eye-specific preference for biological motion

In experiment 1, trials in which the monitors were positioned at 65 deg elicited the highest number of responses. Moreover, we observed the greatest number of responses in the ALE treatment when the stimuli were located at ±50 deg. For the PLE treatment, we observed peaks in responses at ±60 deg and ±180 deg (for the full description, see Results and Supplementary Materials and Methods, ‘SM1 Analysis’; Fig. 3). Consequently, we positioned monitors at 65 deg for experiment 2, with the spider oriented towards the monitors, thereby covering both the ALE and PLE fields of view, and most importantly the crossing point between the two.

Each subject (*n*=179) was assigned to the ALE+PLE (*N*=61), ALE (*N*=58) or PLE (*N*=60) treatment. The spider then underwent two different conditions, across two trials performed on the same day (*M. semilimbatus* show similar response rates between trials when administered the same day, but lower responses when tested a day after; Ferrante et al., 2023 preprint). In the first condition, the spider was positioned on the spherical treadmill and allowed to habituate for 210 s. Then, two stimuli – a random point-light display and a biological point-light display – appeared at ±90 deg and moved towards the center of the screen. These stimuli were identical to those used in our previous experiment (De Agrò et al., 2021) and simply consist of multiple dark ‘pixels’ moving as though in a cloud, with pixel movement determined by stimulus type. The two stimuli proceeded with the same speed and maintained the same angular position relative to each other in each frame. They then paused at ±50 deg for 1 s before resuming movement until they disappeared near the contact point of the two monitors (point of disappearance: ±10 deg). The second condition followed the same procedure as the first, but the stimuli presented were a moving spider silhouette and an equally sized ellipse (also taken from De Agrò et al., 2021). The order of these two conditions was randomized for each spider.

After each stimulus pair, there was a pause of 25 s before a second pair appeared, for a total of 20 repetitions per trial. For each repetition, the position (left/right) of the two stimuli was randomized, as well as the movement direction (either both moving inward, from ±90 deg towards ±10 deg, or outwards, from ±10 deg towards ±90 deg).

As this experiment followed largely the same procedure as our previous work (De Agrò et al., 2021), we followed the same scoring process. In brief, after selecting all *z*-axis peaks, we changed their sign according to the biological stimulus position. This way, rotations in the direction of the biological (or silhouette) stimulus were set as positive values, while rotations in the direction of the random (or ellipse) stimulus were set as negative. All peaks were then included in the analysis, to compute a general pivot tendency. If the spiders performed an equal number of pivots towards the biological and the random stimulus, this would result in an average approaching 0. Likewise, an average >0 would correspond with a preference for the biological stimulus/silhouette, while an average <0 would correspond with a preference for the random stimulus/ellipse. To confirm the validity of this scoring procedure, we also applied it to the results of experiment 1, where only one stimulus at a time was available. With only a single target present, the spiders were expected to turn towards it, resulting in an average significantly and consistently higher than 0.

### Statistical analysis

All analyses were performed using R 4.2.1 (http://www.R-project.org/), including the libraries *readODS* (https://CRAN.R-project.org/package=readODS), *glmmTMB* (Brooks et al., 2017; https://CRAN.R-project.org/package=glmmTMB), car (Fox and Weisberg, 2019), *DHARMa* (https://CRAN.R-project.org/package=DHARMa), *emmeans* (https://CRAN.R-project.org/package=emmeans), *ggplot2* (https://CRAN.R-project.org/package=ggplot2) and *reticulate* (https://CRAN.R-project.org/package=reticulate). Graphical outputs were produced using Python 3 (Van Rossum and Drake, 2009), with the packages *matplotlib* (Hunter, 2007) and *seaborn* (https://zenodo.org/records/8393472).

We employed generalized linear models in our analysis. For each model, we included subject identity as a random intercept and experimental condition as a random slope – as different subjects could have both a different base reactivity (intercept) and a differential response to the conditions (slope). However, this resulted in over-fitting in some cases, which prompted us to remove condition as a random slope. For experiment 1, we modeled the pivot probability as influenced by treatment (ALE, PLE) and monitor setup (120 deg, 65 deg) using a binomial error structure. Regarding the angle of first detection, we plotted the relative frequencies of rotation against the angle of the stimulus and derived the section of highest reactivity. For experiment 2, we modeled the *z*-rotation speed as influenced by treatment (ALE, PLE, ALE+PLE) and condition using a Gaussian error structure.

Below, we report only the main findings. For the full analysis and raw data please refer to Datasets 1–4 and Supplementary Materials and Methods.

## RESULTS

### Experiment 1 – identification of eye-specific visual angles

As anticipated, we observed a higher response probability for the 65 deg screen orientation versus the 120 deg orientation (GLMM *post hoc*, Bonferroni corrected; odds ratio=2.75, s.e.=0.547, *t*=5.084, *P*<0.0001), with no significant difference between ALE and PLE treatments (odds ratio=2.28, s.e.=1.154, *t*=1.629, *P*=0.207).

When considering the position of the stimulus upon pivot initiation, there was a clear peak in responses at around ±50 deg for the ALE treatment (Fig. 3). This is consistent with published values for other species (Zurek and Nelson, 2012a), and suggests the total visual span of the ALEs is ∼100 deg. For the PLE treatment, when the animals were oriented forwards, the majority of responses occurred at around ±60 deg. When the animals were oriented backwards, most responses occurred at ±180 deg (where the stimuli appeared or disappeared at the edge of the monitor). These observations suggest that the PLEs have a wide visual field, from the end of the ALE range on one side (±60 deg), all the way around the back of the animal to the edge of the ALE range on the other side, for a total of ∼260 deg.

Regarding the validity of the scoring procedure for experiment 2, as tested on the data from experiment 1, we observed a significant preferential turning direction towards the stimulus position in the ALE treatments (GLMM *post hoc*, Bonferroni corrected; estimated mean±s.e. 16.306±2.29, *t*=7.128, *P*<0.0001), while there was no such preference in PLE-only spiders (estimated mean±s.e. 3.533±2.34, *t*=1.511, *P*=0.5231). This second result was surprising to us, as the pivot clearly depends on the stimulus position (see Fig. 3), and as such should be directed to the stimulus side, as we qualitatively observed. This may have been due to the lower response rate for the PLE treatment (response rate for ALE treatment: 20.4% versus PLE treatment: 11.1%), which combined with the low sample size may have brought the effect under the significance level. Moreover, in the PLE condition, especially for spiders facing forwards, the stimuli are outside the PLE visual fields for a long time, accumulating a lot of motion independent from detection, contributing to a decrease in the signal-to-noise ratio.

### Experiment 2 – eye-specific preference for biological motion

**Fig. 4.**
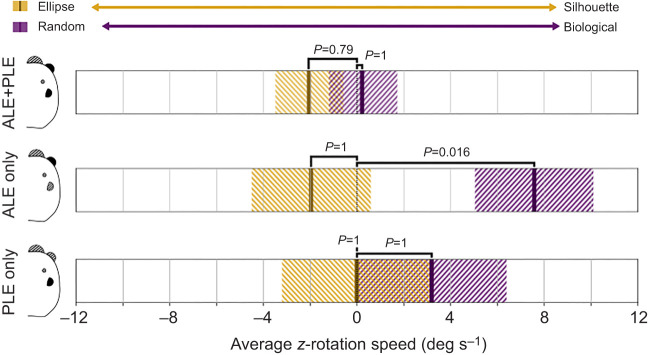
**Results of experiment 2.** The average rotational speed of the sphere *z*-axis is reported (*x*-axis). Negative numbers represent rotations consistent with the position of the non-biological stimulus (ellipse, gold; random, purple), while positive values are rotations consistent with the position of the biological stimulus (silhouette, gold; biological, purple). Dark bars represent the mean; the striped region is the s.e.m. We observed no rotational preference for either the ellipse or the silhouette in any condition. However, spiders were more prone to rotate towards the biological stimulus rather than the random stimulus in the ALE-only condition.

The results of experiment 2 are summarized in Fig. 4. Spiders in the ALE+PLE treatments showed no significant preference for either stimulus in the point-light display (random versus biological) pair (GLMM *post hoc*, Bonferroni corrected; estimated mean±s.e. 0.218±1.44, *t*=0.151, *P*=1) or in the shape (silhouette versus ellipse) pair (estimated mean±s.e. −2.0592±1.37, *t*=−1.506, *P*=0.7926). However, spiders in the ALE treatment showed a significant preference for the biological stimulus in the dots condition (estimated mean±s.e. 7.5723±2.52, *t*=3.003, *P*=0.0161) but no preference in the shapes condition (estimated mean±s.e. −1.9663±2.32, *t*=−0.849, *P*=1). Lastly, spiders in the PLE treatment showed no significant preference for either stimulus in the dots condition (estimated mean±s.e. 3.1869±3.17, *t*=1.004, *P*=1) or in the shapes condition (estimated mean±s.e. −0.0123± 2.55, *t*=−0.005, *P*=1).

## DISCUSSION

In this study, we examined the capacity of jumping spiders to discriminate biological motion point-light displays from random ones. We conducted these tests under partial blindness conditions to investigate whether such discriminatory abilities are specific to a particular eye-pair.

We observed that the spiders were able to discriminate between random and biological displays with the ALEs unblocked, preferring the latter stimulus. However, no preference was observed when only the PLEs were unblocked. This validates our initial hypothesis, suggesting that the circuitry responsible for biological motion recognition is located in ALE-specific visual areas, attesting to the deep specialization of the modular visual system of jumping spiders.

We propose that one of the early, ALE-specific brain areas is responsible for the detection of biological motion, functioning as a low level filter. Neurons fire only when detecting local coherent motion, such as in biological point-light displays. If so, stimuli with fully incoherent local motion (i.e. random point-light-displays) would go completely undetected by the ALEs, with no neuronal firing carrying through to subsequent brain areas. Therefore, in the ALE treatment, spiders exclusively detected the biological motion stimulus and consistently pivoted towards it, as though the biological motion were the only stimulus present. In contrast, the PLEs seem to act as simple motion detectors, with the relevant neural responses registering any translating stimulus. Thus, in the PLE treatment, as the two point-light displays translate across the screen at the same speed and with the same total motion amount, they appeared identical to the spider, conveying equal information to subsequent brain areas. This lack of apparent difference resulted in no preference in the PLE treatment.

Importantly, in our previous experiment (De Agrò et al., 2021) where all the eyes were unblocked, spiders turned more towards the random displays rather than towards the biological ones. The reverse choice observed in the ALE condition suggests multi-eye interaction to be particularly important in informing the spiders' behavior. With all eyes available, a translating biological point-light display smoothly moves across the full field of view of the spider, starting from the PLEs and then passing over the ALE. As the two eye-pairs are equally capable of detecting the stimulus, no mismatch is detected in the switch between the fields. If, however, the moving stimulus is a random point-light display, this will be detected by the PLEs, but will then unexpectedly disappear entering the ALEs. This abrupt information mismatch may violate the spider's ‘expectation’, causing an attention shift towards the vanished object and registering as a preference for the stimulus in our experiment (De Agrò et al., 2021). It is essential to note that by using the term ‘expectation’, we do not imply the necessity of a high-order representation of the object in the spider brain. This behavior can be instead produced with just three neuronal layers, fully fitting in the early areas of the spiders' visual system. We provide a possible organization of such a system in the Appendix. There is indeed a wealth of evidence that shows that spiders maximally produce pivots upon unexpected changes in stimulus behavior, whether it be when a stimulus initially enters their field of view, stops, starts moving, leaves or changes direction (De Agrò et al., 2021).

In the current study, however, the spiders did not show the same preference for random over biological displays in the ALE+PLE condition. We believe there are two possible explanations for the lack of preference. In the current experiments, the two computer monitors were placed at 65 deg to each other, with the two point light displays moving between ±90 deg and ±10 deg. This is in contrast with our previous study, where the stimuli moved between ±60 deg and ±5 deg. This means that in the current experiment, the stimuli spent a much longer time passing across the PLE field only (from ±90 deg to ±60 deg), leaving a long time for the spiders to pivot before gaining information from the ALEs. In our previous experiment (De Agrò et al., 2021), instead, the stimuli just barely appeared in the PLE field, maximizing the importance of the PLE/ALE switch and amplifying the difference. A second explanation may be associated with the unavailability of the AMEs. As previously stated, pivots made towards random displays may be fundamentally an information-seeking effort, directed towards a stimulus that violated expectations. Without AMEs, such a pivot would become redundant – it would bring the random stimulus directly in front of the spider, but this would only move it in the field of view of the ALE, rather than adding any fundamentally new information. It is noteworthy that this behavior would still increase the information intake in the ALE-only and PLE-only conditions, as moving the detected target to the center of the ALE field would at its minimum provide data about its distance (due to the overlap of fields). Nevertheless, further investigation is needed to determine which of these two explanations is the most appropriate, and replication is essential to confirm this is not a Type II error; that is, spiders were capable of discriminating between the two stimuli, but our observation failed to capture it because of low statistical power.

We found that the spiders did not discriminate – that is, they showed no preference – between the silhouette and the ellipse. This outcome aligns with our expectations, considering that the dissimilarity between the two stimuli is primarily shape based, making it more likely to be interpreted by AMEs. Indeed, even though the spider silhouette contains biological motion information, this is much less evident, as the absence of contrast and depth in the image hides the position of the leg joints – information that is instead enhanced in point-light display stimuli. It has been argued that spiders' ALEs could also be capable of discriminating basic shapes, as their resolution should be sufficient for this task (Goté et al., 2019; Zurek et al., 2010). Behaviorally, however, this remains uncertain. Bruce et al. (2021), for example, tested the effect of a distractor appearing in the ALE field during AME scanning of a target stimulus. While the shape of the target influenced the probability of gaze shift, only the motion and not the shape of the distractor had an effect. Spatial acuity alone does not suffice for shape recognition, as it requires dedicated circuitry that may be instead specific to AMEs, following our specialization hypothesis. Although our experiment points in this direction, future studies will be essential to directly test the ability of ALEs regarding shape discrimination.

In this experiment, we unveiled another component of the profound specialization within the jumping spiders' visual system, by revealing distinct roles for individual eye-pairs. How each eye achieves recognition of moving visual patterns as complex as biological displays still remains an open question. We suggested the use of cues such as local coherency, but future studies will be necessary to directly verify the mechanisms behind this ALE specialization, by employing specifically designed stimuli. Moreover, we provided a testable hypothesis for how the interaction between different eye-pairs may determine decision making, increasing the amount of information that each specialized pair can provide. We contend that shifting complex computation upstream and capitalizing on motion-pattern mismatches represents a unique solution to the challenge of brain miniaturization, offering a potential avenue for achieving high performance with limited resources.

### Appendix

## Computational model of jumping spiders' pivoting behavior

**Fig. A1.**
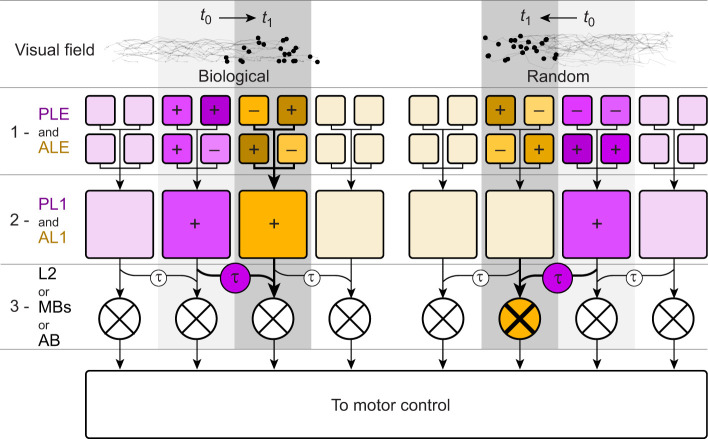
**Proposed computational model of multi-eye integration for the perception of biologically moving stimuli.** PLE, posterior lateral eyes; ALE, anterior lateral eyes; PL1, first neuropil of the PLE; AL2, first neuropil of the ALE; L2, second neuropil of both ALE and PLE; MBs, mushroom bodies; AB, arcuate body. During stimuli motion, ALE and PLE retinas collect light information (1). The pattern of activation is sent to PL1 and AL1 neurons, which produce a signal if the stimuli observed are globally or locally coherent, respectively (2). In the integratory area, the activation of PL1 and AL1 neighboring neurons is compared via delayed activation τ (3). When an object previously detected by AL1 or PL1 moves over a neighboring receptive field, the XOR gate in 3 does not carry the signal over. However, when a translating object can be detected by only one of the eye networks (i.e. random motion by PL1), it is perceived to disappear when in the ALE field, triggering the XOR gate activation in 3 and carrying over the signal to motor control, producing a pivot.

This hypothesized model attempts to account for the observed behavior of jumping spiders, switching preference for producing pivot towards biological versus random motion depending on the available eyes. The computational model is organized across three successive layers (Fig. A1, numbered rows, 1–3). Layer 1 acts as the input layer, and is composed of photosensitive cells; in the jumping spiders' visual system, this would correspond to the eyes (PLEs and ALEs specifically, in the context of this experiment). Layer 2 cells collect input from multiple photoreceptors and extract motion types; in the jumping spiders' visual system, these would be located in the early, eye-specific visual areas (AL1 for ALEs, PL1 for PLEs; for a full description of the visual system organization, see Steinhoff et al., 2020). As per our hypothesis, these cells should be sensitive to specific types of motion: locally coherent for AL1, global direction for PL1. Layer 3 contains cells acting as exclusive or (XOR) gates, receiving direct input from a cell in layer 2, and delayed input from the neighboring ones; in the jumping spiders' visual system, this would be located in a brain region receiving input from both AL1 and PL2 (e.g. the mushroom bodies, the arcuate body, L2). The direct and delayed connections presented here are a very similar system to Hassenstein–Reichardt detector-based systems (Haag et al., 2004; Reichardt, 1987), which describe how motion direction is encoded by the brain. The difference here is that rather than being directly connected to photoreceptors, the comparator and delayed connections are attached to a subsequent visual area. Purple boxes represent cells specific to PLEs and connected areas, while gold boxes represent cells specific to ALEs and connected areas. Brightly colored blocks represent active cells.

As in our experiment, let us assume that across the visual field of the spider, two stimuli are moving, from the side towards the center. On the left there is a biological point-light display, while on the right there is a random one. At time *t*_0_ (light gray background), the two stimuli are moving across the edge of the PLE field. The PLE photoreceptors (layer 1) will react to changes in luminance on the visual field and send signals to the subsequent brain areas. We hypothesize that the PLE visual stream is dedicated to global motion detection. As such, neurons in the dedicated brain region of these eyes (layer 2, PL1) will collect the pattern of activation of photoreceptors and react to the presence of the moving point-cloud. This will happen for both the biological and the random displays.

The stimuli will then continue to move, reaching the start of the ALE field of view at time *t*_1_. ALE photoreceptors (layer 1) will activate as well and send a signal to their dedicated brain region (layer 2, AL1). Crucially, AL1 neurons may be directly tuned for local motion coherency, and will react only for the biological motion pattern, but not for the random one. All neurons of AL1 and PL1 then will project to layer 3. Until a stimulus follows a predictive path, the XOR gates will not activate, as they will receive both the delayed signal of neurons attending to the stimulus position in *t*_0_, and the direct signal from the neurons attending to the stimulus position in *t*_1_. However, as AL1 neurons do not fire for random motion, the signal will not carry over to the dedicated XOR gate, activating it as a result of a mismatch with the delayed connection and sending a signal to the motor control. Pivot direction can be decided according to the relative activation of all the XOR neurons, turning towards the highest firing location. Note that this circuit can also account for stimuli appearing in the visual field, with the XOR gate receiving a signal from the direct connection but no signal from the delayed one (as no activation occurred at the previous time step). The same is true for stimuli changing direction. Lastly, the system can also account for our PLE-only condition: both XOR gates connected to the two locations where the stimuli appeared will equally send signal to motor control, causing pivots to either direction randomly.

## Supplementary Material

10.1242/jexbio.247061_sup1 Supplementary information

Dataset 1. Raw data for experiment 1, expressed as spider rotations as a function of stimulus position

Dataset 2. Raw data for experiment 1, expressed as peak velocities per direction. This is intended as a control to validate the experiment using the same scoring procedure of experiment 2.

Dataset 3. Raw data of experiment 2, first year of testing

Dataset 4. Raw data of experiment 2, second year of testing

Supplementary Materials and Methods SM1 Analysis
