# Supplementary material for: Eye-specific detection and a multi-eye integration model of biological motion perception
Source: J Exp Biol. 2024 Jun 26;227(12):jeb247061. doi: 10.1242/jeb.247061 (PMC11418026; doi:10.1242/jeb.247061)
Supplement: Supplementary information [file jexbio-227-247061-s1.pdf]

Dataset 1. Raw data for experiment 1, expressed as spider rotations as a function of stimulus position

Available for download at
https://journals.biologists.com/jeb/article-lookup/doi/10.1242/jeb.247061#supplementary-data

Dataset 2. Raw data for experiment 1, expressed as peak velocities per direction. This is intended as a control to validate the experiment using the same scoring procedure of experiment 2.

Available for download at
https://journals.biologists.com/jeb/article-lookup/doi/10.1242/jeb.247061#supplementary-data

Dataset 3. Raw data of experiment 2, first year of testing

Available for download at
https://journals.biologists.com/jeb/article-lookup/doi/10.1242/jeb.247061#supplementary-data

Dataset 4. Raw data of experiment 2, second year of testing

Available for download at
https://journals.biologists.com/jeb/article-lookup/doi/10.1242/jeb.247061#supplementary-data

Supplementary Materials and Methods

SM1 Analysis

Available for download at
https://journals.biologists.com/jeb/article-lookup/doi/10.1242/jeb.247061#supplementary-data
